# Supplementary material for: A combined cryo-EM and molecular dynamics approach reveals the mechanism of ErmBL-mediated translation arrest
Source: Nat Commun. 2016 Jul 6;7:12026. doi: 10.1038/ncomms12026 (PMC4935803; doi:10.1038/ncomms12026)
Supplement: Supplementary Information — Supplementary Figures 1-10, Supplementary Table 1 and Supplementary References [file ncomms12026-s1.pdf]

Supplementary Figures

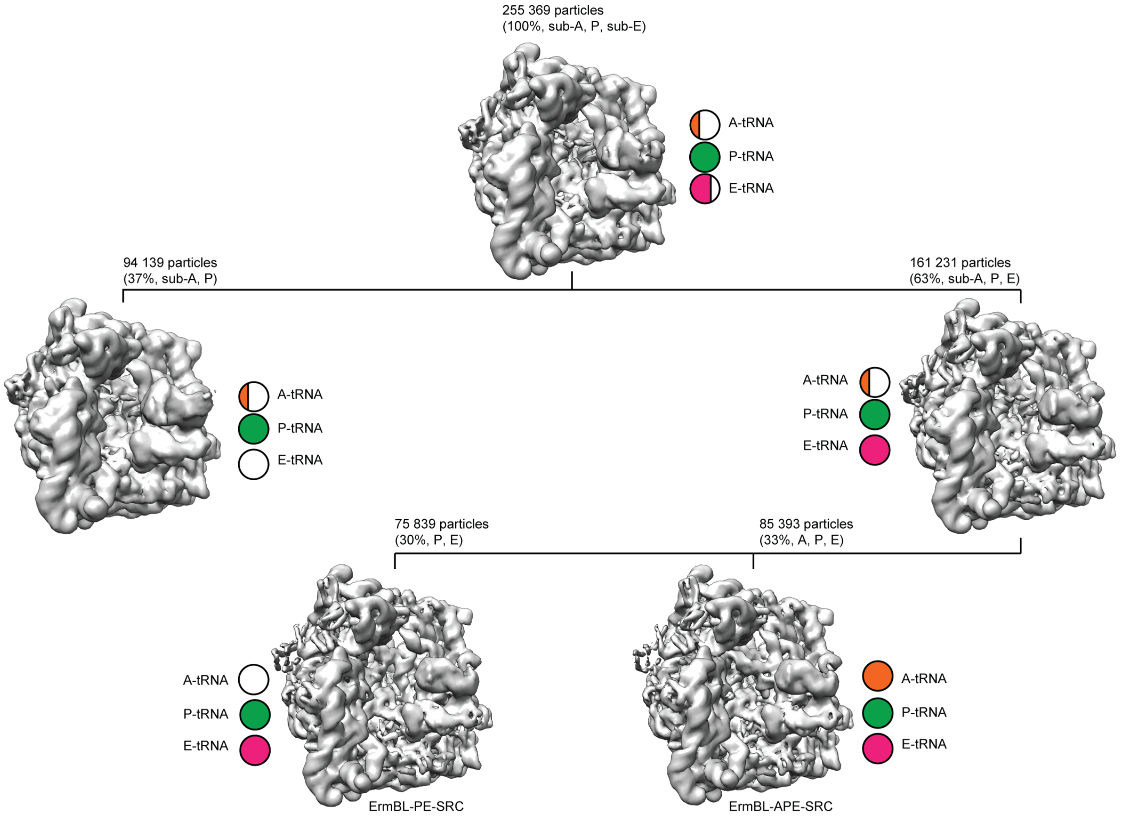

Supplementary Figure 1. *In silico* sorting scheme of the ErmBL-SRC cryo-EM dataset. After removal of non-aligning and edge particles, sorting of the dataset yielded two homogenous sub-datasets. The first (30%; 75,839 particles in total) contained stoichiometric density for P- and E-tRNAs (ErmBL-PE-SRC) and the second (33%; 85,393 particles in total) contained stoichiometric density for A-, P- and E-tRNAs (ErmBL-APE-SRC).

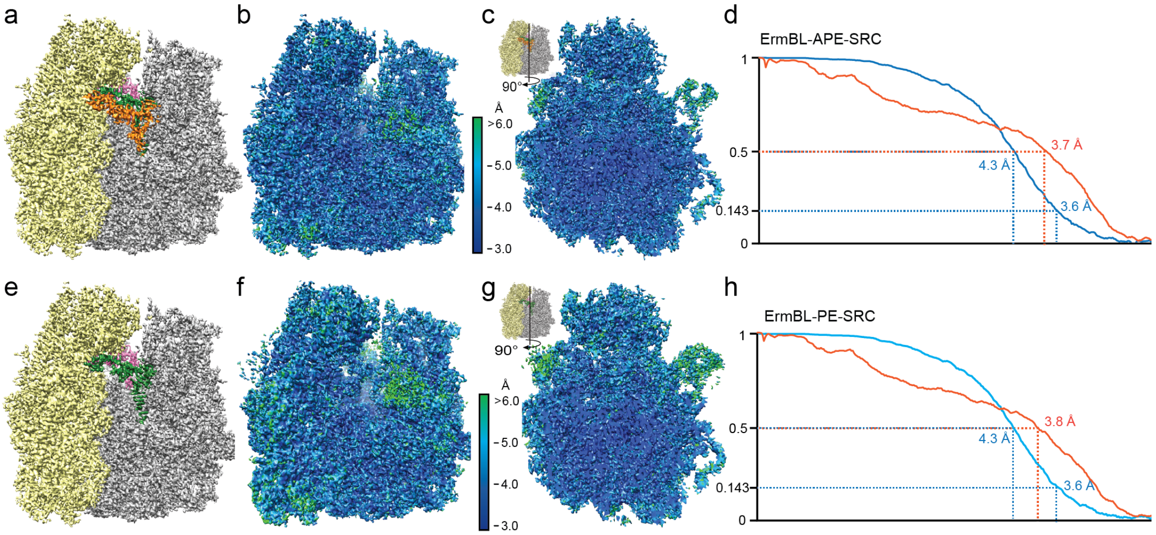

Supplementary Figure 2. Average and local resolution determination of ErmBL-SRCs (**a-d**) Cryo-EM reconstruction of (**a**) ErmBL-APE-SRC bearing A-tRNA (orange), P-tRNA (green) and E-tRNA (pink) and (**b**) the surface or (**c**) the transverse section coloured according to local resolution. (**d**) Average resolution (blue line) of the ErmBL-APE-SRC was 4.3 Å using the Fourier shell correlation (FSC) cut-off value of 0.5. Due to image processing in the absence of spatial frequencies higher than 8 Å, the FSC value of 0.143 was used for average resolution determination of 3.6 Å[1]. The overall fit of the 50S model to the ErmBL- APE-SRC map is indicated by the FSC calculation between the model and map (red curve). (**e-h**) Cryo-EM reconstruction of (**e**) ErmBL-PE-SRC bearing A-tRNA (orange), P-tRNA (green) and E-tRNA (pink) and (**f**) the surface or (**g**) the transverse section coloured according to local resolution. (**h**) Average resolution of the ErmBL-PE-SRC was 4.3 Å using the Fourier shell correlation (FSC) cut-off value of 0.5. Due to image processing in the absence of spatial frequencies higher than 8 Å, the FSC value of 0.143 was used for average resolution determination of 3.6 Å[1]. The overall fit of the 50S model to the ErmBL-PE-SRC map is indicated by the FSC calculation between the model and map (red curve).

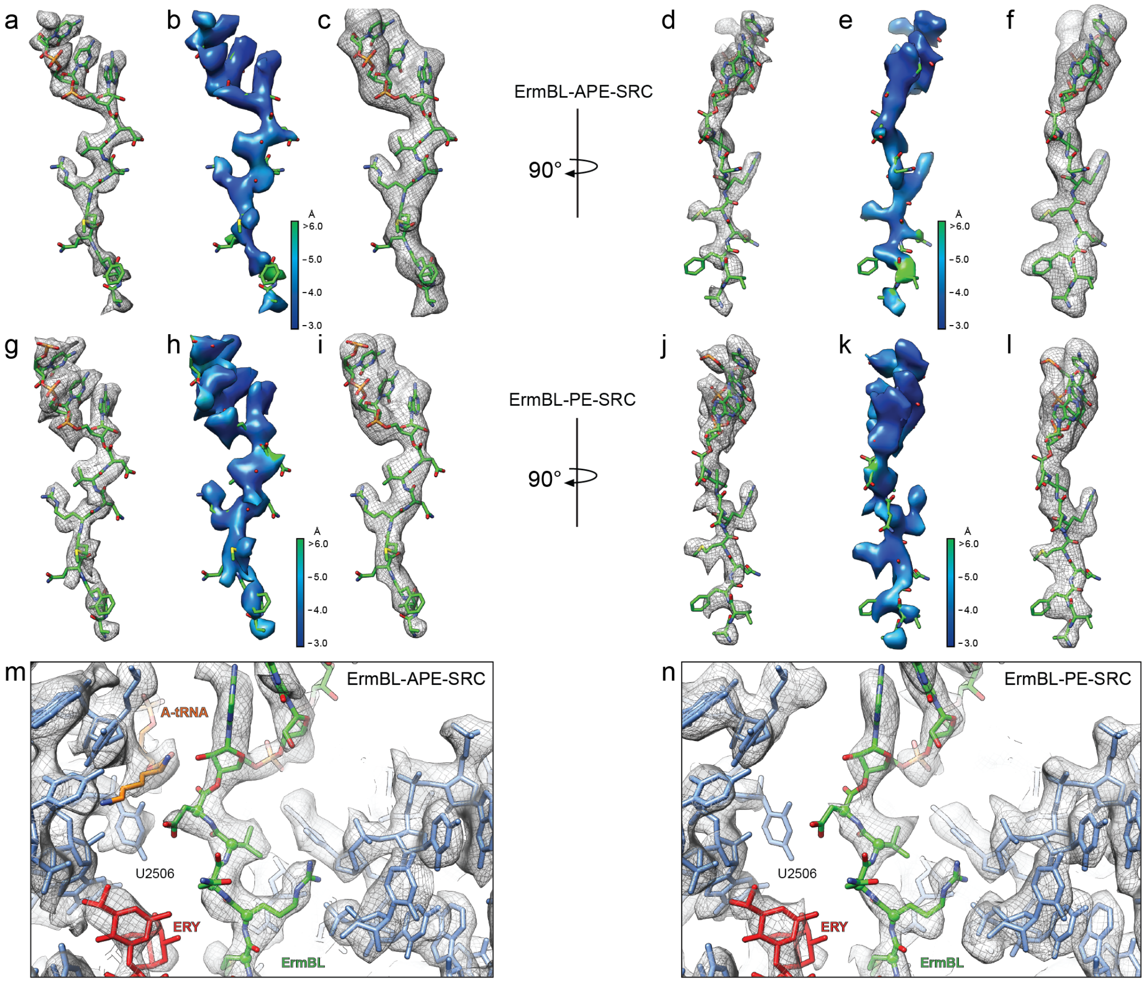

Supplementary Figure 3. Cryo-EM densities for the ErmBL nascent chain in both ErmBL-SRCs (**a-l**) Isolated cryo-EM densities with atomic model for ErmBL nascent chains (green) from (**a-f**) ErmBL-APE-SRC and (**g-l**) ErmBL-PE-SRC with electron density (**b,e,h,k**) coloured according to local resolution or (**c,f,i,l**) filtered to 4-5 Å. (**m,n**) cryo-EM densities (grey mesh) with atomic model for ErmBL-tRNA in the P-site (green), erythromycin (red) and 23S rRNA (blue) from (**m**) ErmBL-APE-SRC including Lys-tRNA in A-site (orange) and (**n**) ErmBL-PE-SRC.

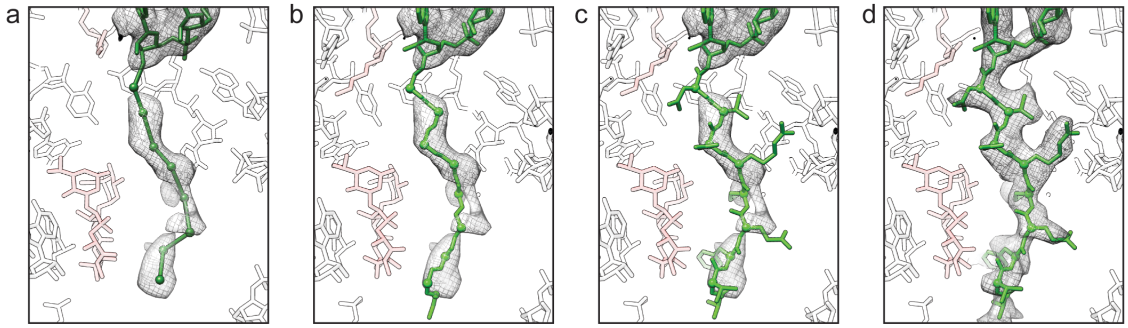

Supplementary Figure 4. Improvement ErmBL-SRC map and model. (**a-c**) Isolated cryo-EM density (grey mesh) of the initial ErmBL-SRC reconstruction (EMD-5771[2]) with Cα backbone trace (**a**) of initially reported for ErmBL nascent chain (PDB 3J5L, green) and (**b**) of the new atomic model and (**c**) including amino acid side chains. (**d**) Isolated cryo-EM density (grey mesh) of the new ErmBL-APE SRC reconstruction including atomic model for ErmBL nascent chain residues V3-D10 (green).

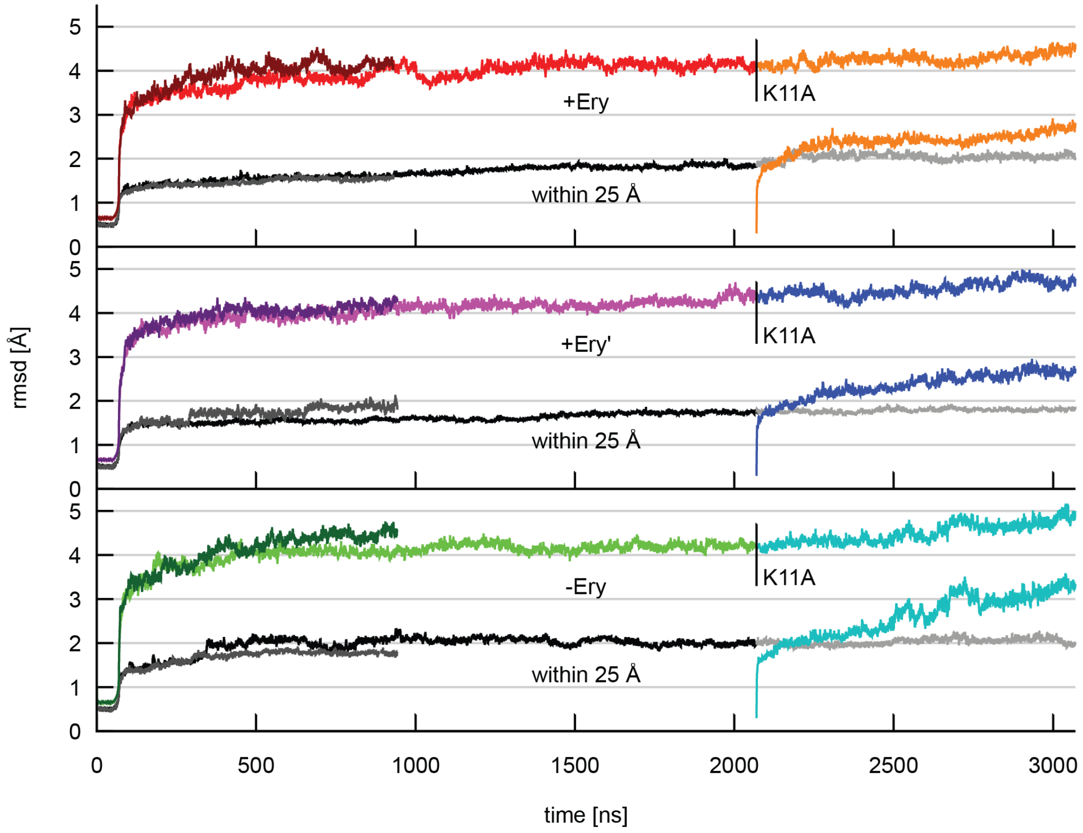

Supplementary Figure 5. Structural deviation from the cryo-EM structure. Root mean square deviation (rmsd) as a function of simulation time for all simulations calculated for the entire ribosome (colored traces) as well as for a subset of atoms within 25 Å of the PTC (Black and grey traces). Top panel: with erythromycin (WT: red, dark red, black and dark grey; Lys11Ala mutation: orange and light grey). Central panel: with erythromycin starting from perturbed Asp10 conformation (WT: magenta, dark magenta, black and dark grey; Lys11Ala: blue and light grey). Bottom panel: without erythromycin (WT: green, dark green, black and dark grey; Lys11Ala: cyan and light grey). In addition, the rmsd from the starting point is shown for the simulations including the Lys11Ala mutation (lower curves in orange, blue and cyan, respectively).

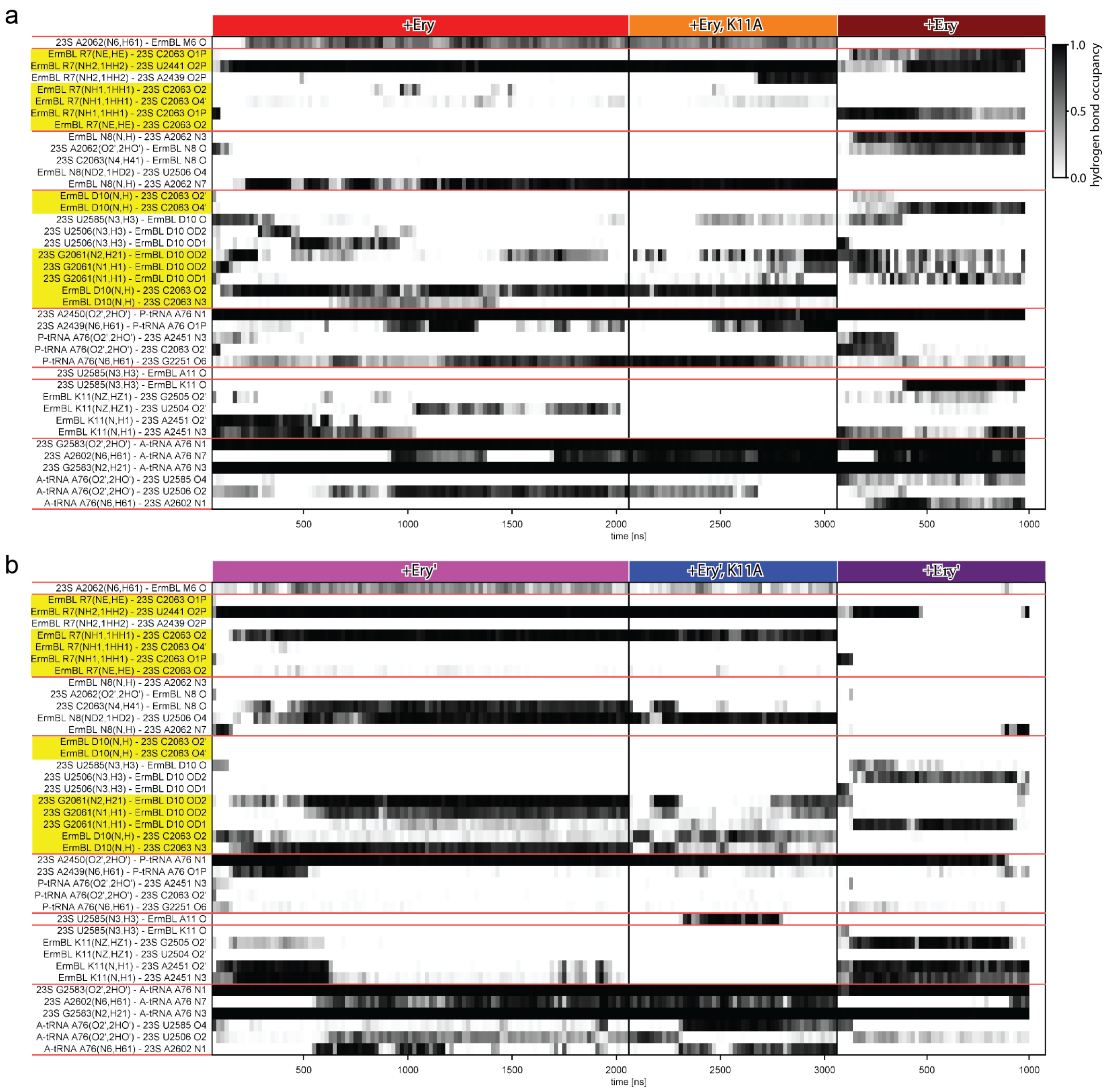

Supplementary Figure 6a,b. Hydrogen bond network between ErmBL peptide and the ribosome. Hydrogen bond occupancies as a function of time for selected hydrogen bonds. Each grey scale block represents the fractional occupancy of the given hydrogen bond calculated over a 20 ns window extracted from the simulations with erythromycin (a, b). Contacts discussed in the main text are highlighted in yellow.

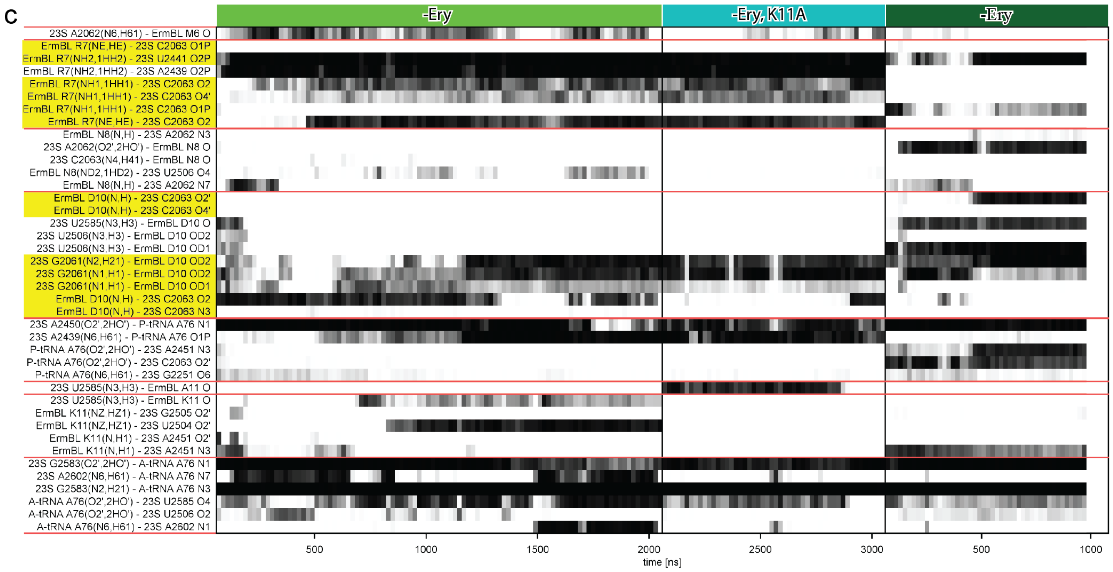

Supplementary Figure 6c. Hydrogen bond network between ErmBL peptide and the ribosome. (c) Hydrogen bond occupancies as a function of time for selected hydrogen bonds. Each grey scale block represents the fractional occupancy of the given hydrogen bond calculated over a 20 ns window extracted from the simulations without erythromycin. Contacts discussed in the main text are highlighted in yellow.

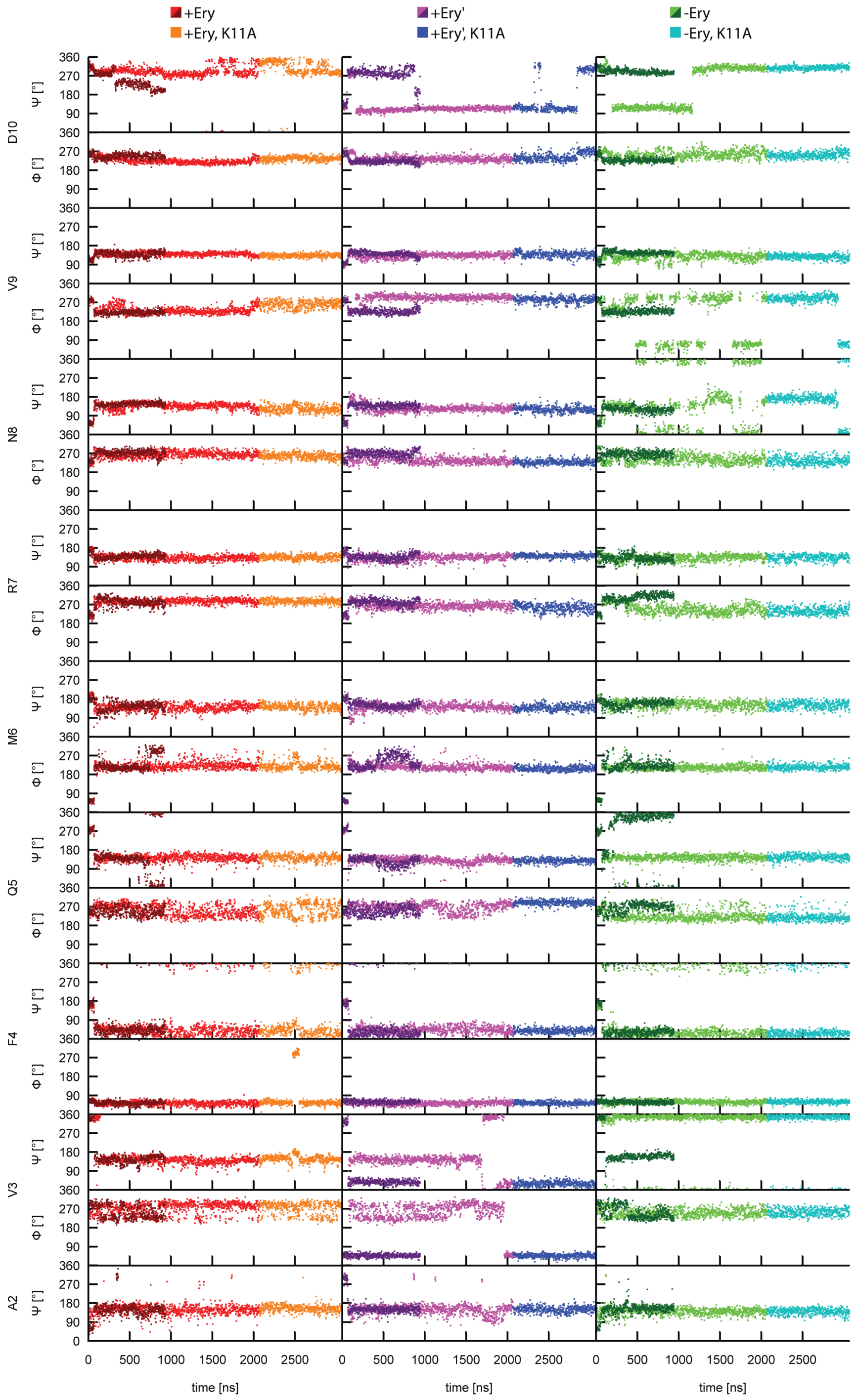

Supplementary Figure 7. Conformation of ErmBL backbone in the course of the simulations. Dihedral angles of all ErmBL amino acids are shown as a function of simulation time for all simulations.

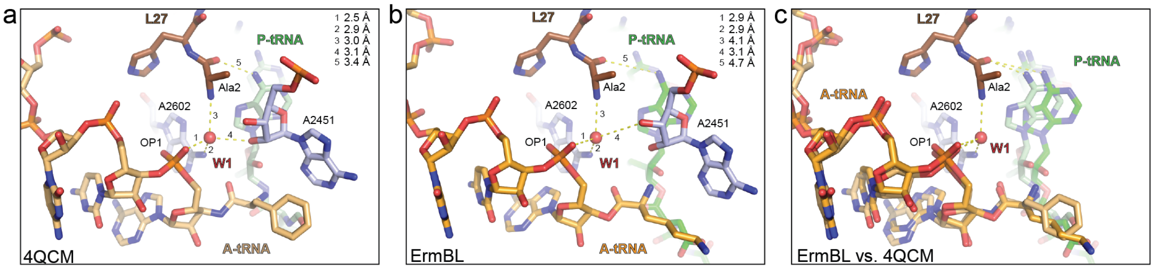

Supplementary Figure 8. Coordination of water W1 during peptide bond formation. (**a-c**) Comparison of the relative position of N-terminus of ribosomal protein L27 (brown), P-tRNA (green), A-tRNA (orange) and 23S rRNA nucleotides A2602 and A2451 (blue) as well as the water molecule W1 (red) in (**a**) pre-attack state (PDB4QCM)[3] and (**b**) ErmBL-SRC. Note that there is no density for A2602 and L27 in the ErmBL-SRC (see **Figure 6h**), therefore the A2602 and L27 positions are superimposed from (a). (**c**) Superimposition of (a) and (b) showing the distance (3.4 Å) between L27 and P-tRNA in (a) increases to 4.7 Å in (b) due to the shifted position of the P-tRNA in the ErmBL-SRC.

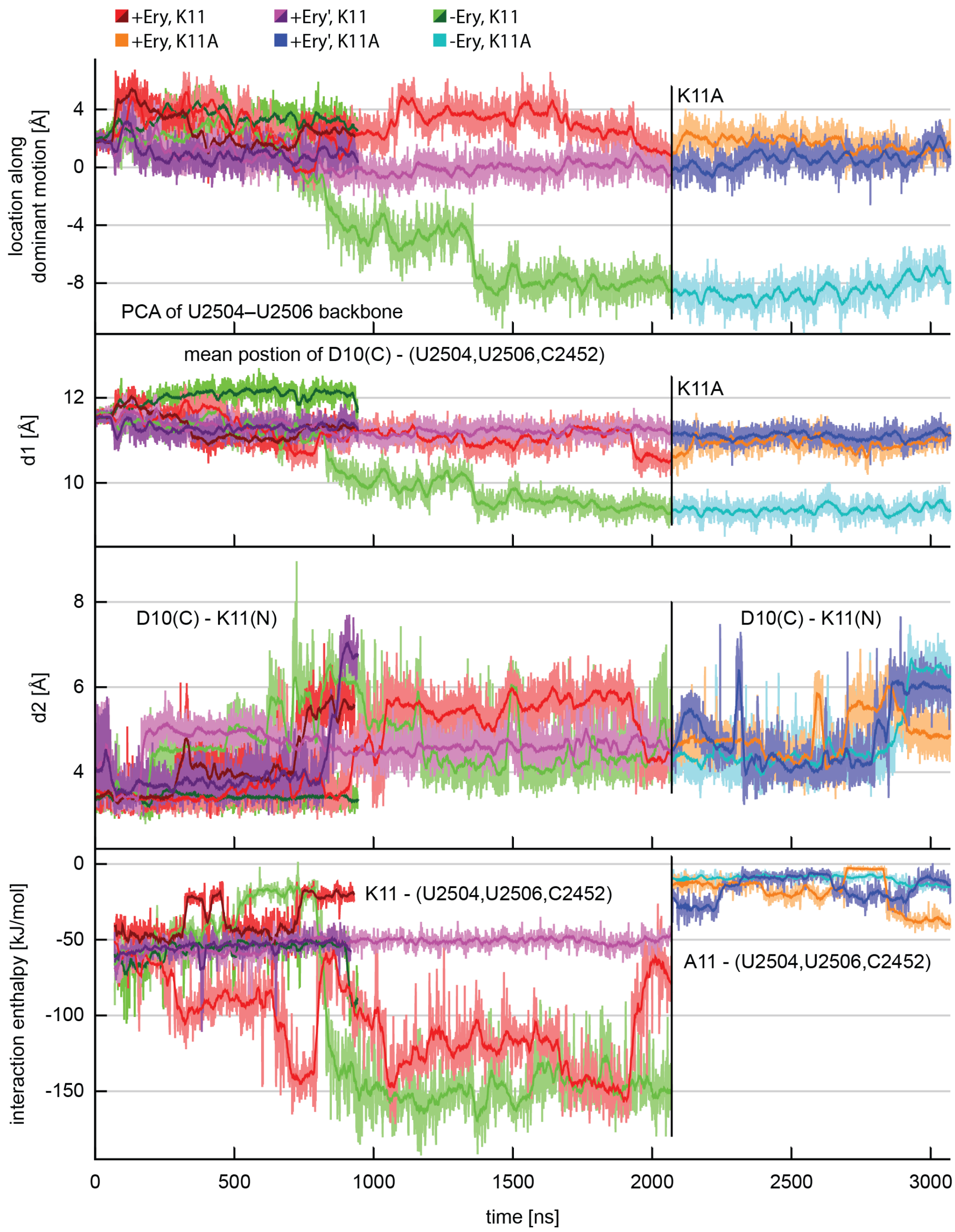

Supplementary Figure 9. The presence of erythromycin influences the conformation of the A-site amino acid. Time traces of the observables used for the histograms in Fig. 7, panels a, c, d, and f.

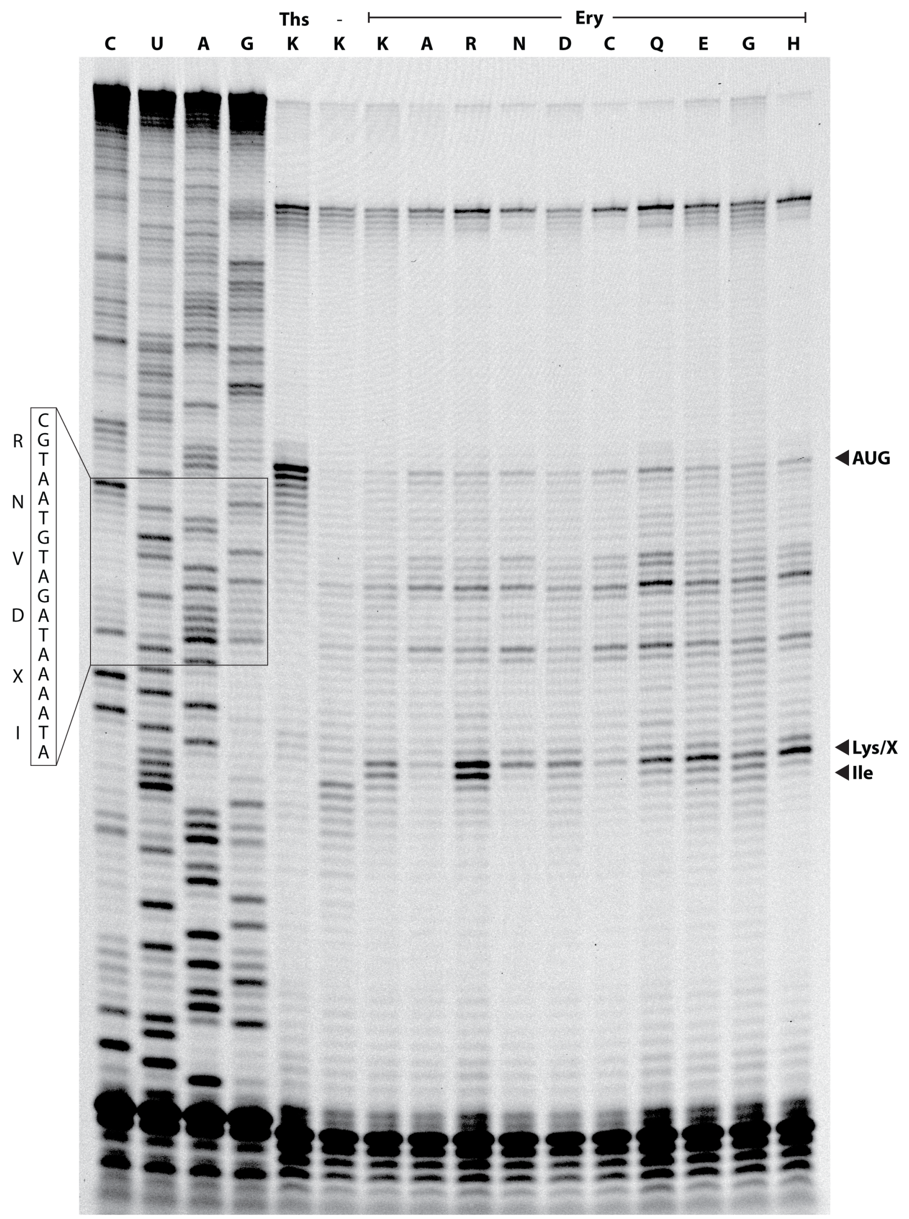

Supplementary Figure 10a. Uncropped toe-print gels for ErmBL mutants. (**a**) Toe-printing assay to monitor translation of ErmBL wildtype in the presence of thiostrepton (Ths), presence or absence (-) of Ery, as well as ErmBL variants (+ Ery) where the Lys11 of ErmBL was changed to all other nineteen sense amino acids, (**a**) Ala-His and (**b**) Ile-Val (on next page). The AUG start codon, the putative stalling site within the RNVDX motif and the Ile catch codon are marked with black arrows on the right hand side.

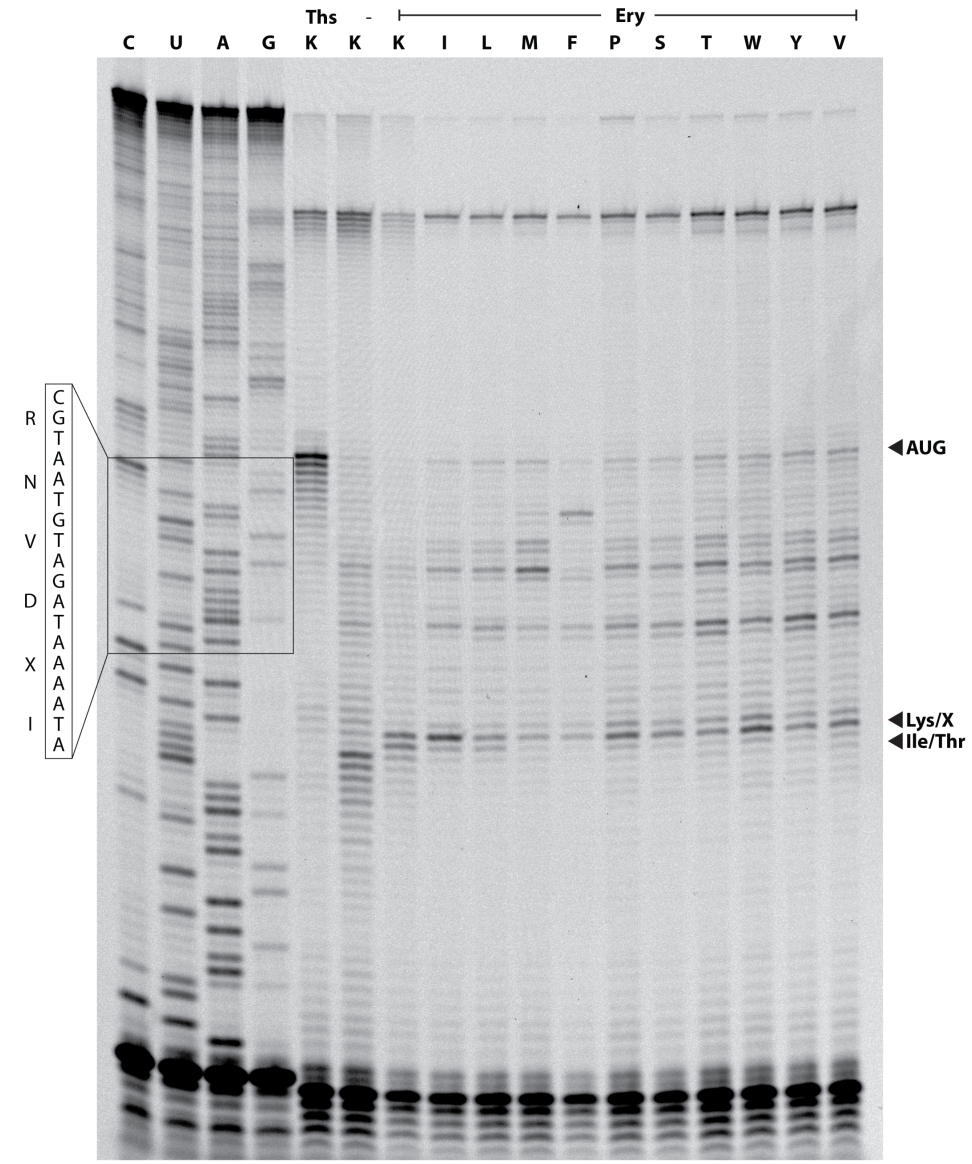

Supplementary Figure 10b. Uncropped toe-print gels. (**a**,**b**) Toe-printing assay to monitor translation of ErmBL wildtype in the presence of thiostrepton (Ths), presence or absence (-) of Ery, as well as ErmBL variants (+ Ery) where the Lys11 of ErmBL was changed to all other nineteen sense amino acids, (**a**) Ala-His (on previous page) and (**b**) Ile-Val. The AUG start codon, the putative stalling site within the RNVDX motif and the Ile catch codon are marked with black arrows on the right hand side.

Supplementary Table 1: Refinement and Model Statistics

**Data Collection and Refinement**	**ErmBL-APE-SRC**	**ErmBL-PE-SRC**
Particles	85,393	75,839
Pixel size (Å)	1.108	1.108
Defocus range (µm)	1.0-2.4	1.0-2.4
Voltage (kV)	300	300
Electron dose (e^-/Å^{-2})	28	28
Map sharpening B factor (Å^2)	-147.40	-140.09
Resolution (Å, 0.143 FSC)	3.6	3.6
**Model Composition**		
Non-hydrogen atoms	146778	145176
Protein residues	5626	5626
RNA bases	4761	4696
**Validation (proteins)**		
Poor rotamers (%)	0.00	0.00
Ramachandran outliers (%)	2.68	2.68
Ramachandran favored (%)	89.95	89.99
Bad backbone bonds (%)	0.00	0.00
Bad backbone angles	0.00	0.00
MolProbity score	1.89 (81st percentile)	1.88 (82nd percentile)
**Validation (nucleic acids)**		
Correct sugar puckers (%)	99.67	99.78
Bad backbone conformations (%)	14.18	14.30
Bad bonds (%)	1.06	1.06
Bad angles	0.00	0.00
**Clashscore, all atoms**	**6.11 (90th percentile)**	**5.99 (90th percentile)**

Supplementary References

1. Scheres, S.H. & Chen, S. Prevention of overfitting in cryo-EM structure determination. *Nat Methods* **9**, 853-4 (2012).
2. Arenz, S. et al. Molecular basis for erythromycin-dependent ribosome stalling during translation of the ErmBL leader peptide. *Nature Communications* **5**, 3501 (2014).
3. Polikanov, Y.S., Steitz, T.A. & Innis, C.A. A proton wire to couple aminoacyl-tRNA accommodation and peptide-bond formation on the ribosome. *Nat Struct Mol Biol* **21**, 787-93 (2014).
